# Ethyl 2-(5-bromo-3-ethyl­sulfinyl-1-benzofuran-2-yl)acetate

**DOI:** 10.1107/S1600536809009775

**Published:** 2009-03-25

**Authors:** Hong Dae Choi, Pil Ja Seo, Byeng Wha Son, Uk Lee

**Affiliations:** a Department of Chemistry, Dongeui University, San 24 Kaya-dong Busanjin-gu, Busan 614-714, Republic of Korea; b Department of Chemistry, Pukyong National University, 599-1 Daeyeon 3-dong, Nam-gu, Busan 608-737, Republic of Korea

## Abstract

The title compound, C_14_H_15_BrO_4_S, was prepared by the oxidation of ethyl 2-(5-bromo-3-ethyl­sulfanyl-1-benzofuran-2-yl)acetate with 3-chloro­peroxy­benzoic acid. The crystal structure is stabilized by aromatic π–π inter­actions between the benzene rings of neighbouring mol­ecules [centroid–centroid distance = 3.814 (9) Å], and possibly by weak C—H⋯π inter­actions. In addition, the crystal structure exhibits three inter­molecular C—H⋯O non-classical hydrogen bonds. The ethyl group bonded to carboxyl­ate O atom is disordered over two positions, with refined site-occupancy factors of 0.686 (18) and 0.314 (18).

## Related literature

For the crystal structures of similar alkyl 2-(1-benzofuran-2-yl)acetate derivatives, see: Choi *et al.* (2008*a* ▶,*b* ▶, 2009 ▶).
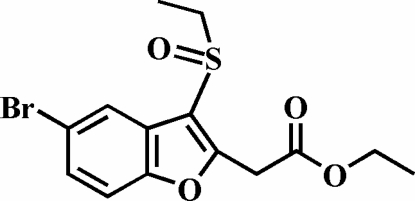


## Experimental

### 

#### Crystal data

C_14_H_15_BrO_4_S
*M* *_r_* = 359.23
Triclinic, 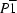

*a* = 8.311 (3) Å
*b* = 9.800 (3) Å
*c* = 10.621 (3) Å
α = 69.552 (5)°
β = 77.671 (6)°
γ = 66.259 (5)°
*V* = 739.3 (4) Å^3^
*Z* = 2
Mo *K*α radiation
μ = 2.93 mm^−1^
*T* = 298 K
0.30 × 0.20 × 0.10 mm

#### Data collection

Bruker SMART CCD diffractometer
Absorption correction: multi-scan (*SADABS*; Sheldrick, 1999 ▶) *T*_min_ = 0.496, *T*_max_ = 0.750
5330 measured reflections
2562 independent reflections
1790 reflections with *I* > 2σ(*I*)
*R*_int_ = 0.042

#### Refinement

*R*[*F* ^2^ > 2σ(*F* ^2^)] = 0.057
*wR*(*F* ^2^) = 0.168
*S* = 1.12
2562 reflections
191 parameters
29 restraints
H-atom parameters constrained
Δρ_max_ = 0.65 e Å^−3^
Δρ_min_ = −0.58 e Å^−3^

### 

Data collection: *SMART* (Bruker, 2001 ▶); cell refinement: *SAINT* (Bruker, 2001 ▶); data reduction: *SAINT*; program(s) used to solve structure: *SHELXS97* (Sheldrick, 2008 ▶); program(s) used to refine structure: *SHELXL97* (Sheldrick, 2008 ▶); molecular graphics: *ORTEP-3* (Farrugia, 1997 ▶) and *DIAMOND* (Brandenburg, 1998 ▶); software used to prepare material for publication: *SHELXL97*.

## Supplementary Material

Crystal structure: contains datablocks global, I. DOI: 10.1107/S1600536809009775/rk2134sup1.cif

Structure factors: contains datablocks I. DOI: 10.1107/S1600536809009775/rk2134Isup2.hkl

Additional supplementary materials:  crystallographic information; 3D view; checkCIF report

## Figures and Tables

**Table 1:** Hydrogen-bond geometry (Å, °)

*D*—H⋯*A*	*D*—H	H⋯*A*	*D*⋯*A*	*D*—H⋯*A*
C12*A*—H12*B*⋯*Cg*2^i^	0.96	2.92	3.85 (1)	164
C3—H3⋯O4^ii^	0.93	2.67	3.556 (8)	160
C5—H5⋯O3^iii^	0.93	2.67	3.528 (9)	155
C9—H9*B*⋯O4^iv^	0.97	2.35	3.277 (9)	161

Symmetry codes: (i) 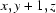; (ii) 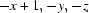; (iii) 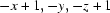; (iv) 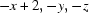. *Cg*2 is the centroid of the C1/C2/C7/O1/C8 furan ring.

**Fig. 1.**
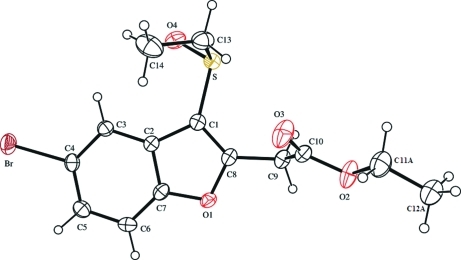
The molecular structure of the title compound with the atom numbering scheme. Displacement ellipsoids are drawn at the 30% probability level. H atoms are presented as a small cycles with arbitrary radius. Only major component (C11A and C12A) is drawn.

**Fig. 2.**
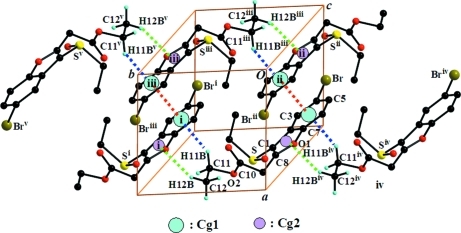
The π–π and C—H···π interactions (dotted lines) in the crystal structure of title compound. Cg denotes the ring centroid. The disordered component of the ethyl group bonded to carboxylate O atom, part B, has been omitted for clairty as have H atoms not involved in intermolecular contacts. [Symmetry codes: (i) x, y+1, z; (ii) x, 1+y, z; (iii) -x+1, -y+1, -z+1; (iv) x, y-1, z; (v) -x+1, -y+2, -z+1].

## supplementary crystallographic information

### Comment

As a part of our continuing studies on the synthesis and structure of alkyl 2-(1-benzofuran-2-yl)acetate analogues, we have recently described the crystal structure of isopropyl 2-(5-bromo-3-methylsulfinyl-1-benzofuran-2-yl)acetate (Choi *et al.*, 2008*a*), methyl 2-(5-bromo-3-methylsulfinyl-1-benzofuran-2-yl)acetate (Choi *et al.*, 2008*b*), and ethyl 2-(3-ethylsulfinyl-5-methyl-1-benzofuran-2-yl)acetate (Choi *et al.*, 2009). Here we report the crystal structure of the title compound, ethyl 2-(5-bromo-3-ethylsulfinyl-1-benzofuran-2-yl)acetate (Fig. 1).

The benzofuran unit is essentially planar, with a mean deviation of 0.009 (5) Å from the least-squares plane defined by the nine constituent atoms. The ethyl group bonded to carboxylate O atom is disordered over two positions with site-occupancy factors of 0.686 (18) (for atoms labelled A) and 0.314 (18) (for atoms labelled B). The molecular packing (Fig. 2) is stabilized by aromatic π–π interactions between the benzene rings of the adjacent molecules, with a Cg1···Cg1^ii^ distance of 3.814 (9) Å (Cg1 is the centroid of the C2–C7 benzene ring; symmetry code as in Fig. 2). The crystal packing is further stabilized by intermolecular C—H···π interactions; a first between the methylene H atom of ethoxy group and the benzene ring of a neighbouring molecule, with C11—H11B···Cg1^i^, a second between the methyl H atom of ethoxy group and the furan ring of a neighbouring benzofuran fragment, with C12—H12B···Cg2^i^, respectively (Table 1 and Fig. 2; Cg2 is the centroid of the C1/C2/C7/O1/C8 furan ring; symmetry code as in Fig. 2). Additionally, the crystal packing exhibits weak intermolecular C—H···O nonclassical hydrogen bonds; a first between a benzene H atom and the S═O unit, a second between a benzene H atom and the C═O unit, a third between an H atom of the methylene group bonded to carboxylate C atom and the S═O unit, respectively (Table 1).

### Experimental

The 77% 3-chloroperoxybenzoic acid (247 mg, 1.1 mmol) was added in small portions to a stirred solution of ethyl 2-(5-bromo-3-ethylsulfanyl-1-benzofuran-2-yl)acetate (343 mg, 1.0 mmol) in dichloromethane (40 ml) at 273 K. After being stirred for 3 h at room temperature, the mixture was washed with saturated sodium bicarbonate solution and the organic layer was separated, dried over magnesium sulfate, filtered and concentrated in vacuum. The residue was purified by column chromatography (hexane–ethyl acetate, 1:2 *v*/*v*) to afford the title compound as a colourless solid [yield 78%, m.p. 391–392 K; *R*_f_ = 0.54 (hexane–ethyl acetate, 1:2 *v*/*v*)]. Single crystals suitable for X-ray diffraction were prepared by evaporation of a solution of the title compound in chloroform at room temperature. Spectroscopic analysis: ^1^H NMR (CDCl_3_, 400 MHz) δ 1.28 (t, J = 6.96 Hz, 3H), 1.33 (t, J = 7.32 Hz, 3H), 3.28 (q, J = 7.32 Hz, 2H), 4.05 (s, 2H), 4.21 (q, J = 6.96 Hz, 2H), 7.40 (d, J = 8.76 Hz, 1H), 7.34 (dd, J = 8.44 Hz and J = 1.84 Hz, 1H), 8.01 (s, 1H); EI–MS 360 [M+2], 358 [M^+^].

### Refinement

All H atoms were geometrically positioned and refined using a riding model, with C—H = 0.93 Å for the aryl, 0.97 Å for the methylene, and 0.96 Å for the methyl H atoms with U_iso_(H) = 1.2U_eq_(C) for the aryl and methylene H atoms, and 1.5U_eq_(C) for methyl H atoms. The ethyl group bonded to carboxylate O atom was found to be disordered over two positions and modelled with site-occupancy factors, from refinement, of 0.686 (18) (C11A–C12A) and 0.314 (18) (C11B–C12B). The both sets of C atoms were restrained using the command SADI(0.02), ISOR(0.01), DELU, EADP and the C—C distances (A & B) were restrained to 1.480 (2) Å using command DFIX.

### Crystal data

**Table:**

C_14_H_15_BrO_4_S	*Z* = 2
*M**_r_* = 359.23	*F*(000) = 364
Triclinic, *P*1	*D*_x_ = 1.614 Mg m^−^^3^
Hall symbol: -P 1	Melting point: 391.5 K
*a* = 8.311 (3) Å	Mo *K*α radiation, λ = 0.71073 Å
*b* = 9.800 (3) Å	Cell parameters from 1919 reflections
*c* = 10.621 (3) Å	θ = 2.4–25.0°
α = 69.552 (5)°	µ = 2.93 mm^−^^1^
β = 77.671 (6)°	*T* = 298 K
γ = 66.259 (5)°	Block, colourless
*V* = 739.3 (4) Å^3^	0.30 × 0.20 × 0.10 mm

### Data collection

**Table:**

Bruker SMART CCD diffractometer	2562 independent reflections
Radiation source: Fine-focus sealed tube	1790 reflections with *I* > 2σ(*I*)
Graphite	*R*_int_ = 0.042
Detector resolution: 10.0 pixels mm^-1^	θ_max_ = 25.0°, θ_min_ = 2.1°
φ and ω scans	*h* = −9→9
Absorption correction: multi-scan (*SADABS*; Sheldrick, 1999)	*k* = −11→11
*T*_min_ = 0.496, *T*_max_ = 0.750	*l* = −12→12
5330 measured reflections	

### Refinement

**Table:**

Refinement on *F*^2^	Primary atom site location: Direct
Least-squares matrix: Full	Secondary atom site location: Difmap
*R*[*F*^2^ > 2σ(*F*^2^)] = 0.057	H-atom parameters constrained
*wR*(*F*^2^) = 0.168	*w* = 1/[σ^2^(*F*_o_^2^) + (0.0738*P*)^2^ + 1.336*P*] where *P* = (*F*_o_^2^ + 2*F*_c_^2^)/3
*S* = 1.12	(Δ/σ)_max_ < 0.001
2562 reflections	Δρ_max_ = 0.65 e Å^−^^3^
191 parameters	Δρ_min_ = −0.58 e Å^−^^3^
29 restraints	

### Special details

**Table:**

Geometry. All s.u.'s (except the s.u. in the dihedral angle between two l.s. planes) are estimated using the full covariance matrix. The cell s.u.'s are taken into account individually in the estimation of s.u.'s in distances, angles and torsion angles; correlations between s.u.'s in cell parameters are only used when they are defined by crystal symmetry. An approximate (isotropic) treatment of cell s.u.'s is used for estimating s.u.'s involving l.s. planes.
Refinement. Refinement of F^2^ against ALL reflections. The weighted R-factor wR and goodness of fit S are based on F^2^, conventional R-factors R are based on F, with F set to zero for negative F^2^. The threshold expression of F^2^ > 2σ(F^2^) is used only for calculating R-factors(gt) etc. and is not relevant to the choice of reflections for refinement. R-factors based on F^2^ are statistically about twice as large as those based on F, and R-factors based on ALL data will be even larger.

### Fractional atomic coordinates and isotropic or equivalent isotropic displacement parameters (Å^2^)

**Table:**

	*x*	*y*	*z*	*U*_iso_*/*U*_eq_	Occ. (<1)
Br	0.31159 (11)	−0.28976 (10)	0.39050 (8)	0.0551 (3)	
S	0.7649 (2)	0.1124 (2)	0.02737 (16)	0.0413 (5)	
O1	0.8405 (6)	−0.0339 (5)	0.4145 (4)	0.0363 (11)	
O2	0.9901 (7)	0.3669 (6)	0.2625 (6)	0.0560 (14)	
O3	0.7412 (8)	0.3819 (6)	0.2043 (7)	0.0694 (17)	
O4	0.7327 (7)	0.0002 (6)	−0.0200 (5)	0.0525 (13)	
C1	0.7580 (8)	0.0383 (7)	0.2055 (6)	0.0330 (15)	
C2	0.6623 (8)	−0.0604 (7)	0.2949 (6)	0.0299 (14)	
C3	0.5405 (9)	−0.1172 (8)	0.2829 (6)	0.0355 (15)	
H3	0.4975	−0.0921	0.2006	0.043*	
C4	0.4856 (9)	−0.2139 (7)	0.4000 (7)	0.0359 (16)	
C5	0.5436 (9)	−0.2537 (8)	0.5241 (7)	0.0403 (16)	
H5	0.5006	−0.3177	0.5992	0.048*	
C6	0.6667 (9)	−0.1981 (8)	0.5368 (6)	0.0412 (17)	
H6	0.7107	−0.2254	0.6192	0.049*	
C7	0.7211 (8)	−0.1005 (8)	0.4222 (7)	0.0352 (15)	
C8	0.8584 (9)	0.0501 (7)	0.2814 (7)	0.0356 (15)	
C9	0.9829 (9)	0.1352 (8)	0.2529 (7)	0.0413 (17)	
H9A	1.0576	0.0907	0.3267	0.050*	
H9B	1.0582	0.1208	0.1716	0.050*	
C10	0.8891 (10)	0.3058 (9)	0.2353 (7)	0.0427 (18)	
C11A	0.9188 (18)	0.5401 (18)	0.2280 (15)	0.063 (4)	0.686 (18)
H11A	0.9056	0.5869	0.1321	0.076*	0.686 (18)
H11B	0.8046	0.5759	0.2768	0.076*	0.686 (18)
C12A	1.0484 (16)	0.5825 (15)	0.2679 (15)	0.062 (4)	0.686 (18)
H12A	1.0704	0.5257	0.3604	0.093*	0.686 (18)
H12B	1.0023	0.6924	0.2575	0.093*	0.686 (18)
H12C	1.1566	0.5569	0.2117	0.093*	0.686 (18)
C11B	0.907 (4)	0.515 (4)	0.288 (3)	0.063 (4)	0.314 (18)
H11C	0.9183	0.5081	0.3793	0.076*	0.314 (18)
H11D	0.7833	0.5622	0.2708	0.076*	0.314 (18)
C12B	1.014 (4)	0.600 (3)	0.187 (3)	0.062 (4)	0.314 (18)
H12D	0.9653	0.6418	0.1013	0.093*	0.314 (18)
H12E	1.1330	0.5299	0.1792	0.093*	0.314 (18)
H12F	1.0114	0.6839	0.2154	0.093*	0.314 (18)
C13	0.5715 (11)	0.2879 (9)	0.0038 (7)	0.053 (2)	
H13A	0.5850	0.3558	0.0456	0.063*	
H13B	0.5662	0.3412	−0.0921	0.063*	
C14	0.3993 (12)	0.2654 (10)	0.0593 (9)	0.069 (3)	
H14A	0.3947	0.1822	0.0339	0.103*	
H14B	0.3039	0.3599	0.0236	0.103*	
H14C	0.3890	0.2397	0.1558	0.103*	

### Atomic displacement parameters (Å^2^)

**Table:**

	*U*^11^	*U*^22^	*U*^33^	*U*^12^	*U*^13^	*U*^23^
Br	0.0555 (6)	0.0564 (6)	0.0633 (6)	−0.0350 (4)	−0.0083 (4)	−0.0095 (4)
S	0.0424 (11)	0.0489 (11)	0.0339 (9)	−0.0210 (9)	−0.0021 (8)	−0.0089 (8)
O1	0.032 (3)	0.044 (3)	0.034 (2)	−0.013 (2)	−0.0099 (19)	−0.011 (2)
O2	0.037 (3)	0.039 (3)	0.101 (4)	−0.009 (2)	−0.016 (3)	−0.030 (3)
O3	0.052 (4)	0.045 (3)	0.109 (5)	−0.010 (3)	−0.045 (3)	−0.006 (3)
O4	0.059 (3)	0.060 (3)	0.046 (3)	−0.019 (3)	−0.007 (2)	−0.025 (3)
C1	0.032 (4)	0.026 (3)	0.038 (4)	−0.006 (3)	−0.005 (3)	−0.009 (3)
C2	0.027 (3)	0.026 (3)	0.035 (3)	−0.005 (3)	−0.004 (3)	−0.012 (3)
C3	0.033 (4)	0.036 (4)	0.034 (4)	−0.010 (3)	−0.006 (3)	−0.007 (3)
C4	0.035 (4)	0.029 (4)	0.045 (4)	−0.013 (3)	−0.002 (3)	−0.012 (3)
C5	0.042 (4)	0.033 (4)	0.039 (4)	−0.011 (3)	−0.003 (3)	−0.006 (3)
C6	0.046 (5)	0.045 (4)	0.029 (4)	−0.013 (4)	−0.005 (3)	−0.009 (3)
C7	0.029 (4)	0.034 (4)	0.043 (4)	−0.006 (3)	−0.007 (3)	−0.016 (3)
C8	0.033 (4)	0.032 (4)	0.044 (4)	−0.010 (3)	−0.008 (3)	−0.013 (3)
C9	0.034 (4)	0.045 (4)	0.050 (4)	−0.015 (3)	−0.007 (3)	−0.017 (3)
C10	0.047 (5)	0.049 (5)	0.041 (4)	−0.025 (4)	−0.012 (3)	−0.010 (3)
C11A	0.066 (6)	0.052 (6)	0.081 (8)	−0.023 (4)	−0.014 (5)	−0.024 (5)
C12A	0.061 (6)	0.057 (5)	0.077 (7)	−0.021 (4)	−0.015 (5)	−0.024 (5)
C11B	0.066 (6)	0.052 (6)	0.081 (8)	−0.023 (4)	−0.014 (5)	−0.024 (5)
C12B	0.061 (6)	0.057 (5)	0.077 (7)	−0.021 (4)	−0.015 (5)	−0.024 (5)
C13	0.070 (6)	0.042 (4)	0.045 (4)	−0.018 (4)	−0.016 (4)	−0.007 (3)
C14	0.065 (6)	0.053 (5)	0.058 (5)	−0.004 (5)	−0.002 (4)	−0.003 (4)

### Geometric parameters (Å, °)

**Table:**

Br—C4	1.907 (6)	C9—C10	1.494 (10)
S—O4	1.487 (5)	C9—H9A	0.9700
S—C1	1.771 (6)	C9—H9B	0.9700
S—C13	1.802 (8)	C11A—C12A	1.480 (2)
O1—C7	1.366 (7)	C11A—H11A	0.9700
O1—C8	1.376 (8)	C11A—H11B	0.9700
O2—C10	1.332 (8)	C12A—H12A	0.9600
O2—C11B	1.43 (4)	C12A—H12B	0.9600
O2—C11A	1.489 (16)	C12A—H12C	0.9600
O3—C10	1.199 (9)	C11B—C12B	1.480 (2)
C1—C8	1.338 (9)	C11B—H11C	0.9700
C1—C2	1.458 (8)	C11B—H11D	0.9700
C2—C3	1.382 (9)	C12B—H12D	0.9600
C2—C7	1.405 (9)	C12B—H12E	0.9600
C3—C4	1.391 (9)	C12B—H12F	0.9600
C3—H3	0.9300	C13—C14	1.499 (11)
C4—C5	1.371 (9)	C13—H13A	0.9700
C5—C6	1.387 (9)	C13—H13B	0.9700
C5—H5	0.9300	C14—H14A	0.9600
C6—C7	1.379 (9)	C14—H14B	0.9600
C6—H6	0.9300	C14—H14C	0.9600
C8—C9	1.498 (9)		
			
O4—S—C1	106.2 (3)	C8—C9—H9B	109.1
O4—S—C13	108.0 (3)	H9A—C9—H9B	107.8
C1—S—C13	101.7 (3)	O3—C10—O2	122.3 (7)
C7—O1—C8	106.1 (5)	O3—C10—C9	126.6 (6)
C10—O2—C11B	118.2 (14)	O2—C10—C9	111.2 (6)
C10—O2—C11A	114.9 (6)	C12A—C11A—O2	106.0 (10)
C11B—O2—C11A	23.6 (12)	C12A—C11A—H11A	110.5
C8—C1—C2	107.1 (6)	O2—C11A—H11A	110.5
C8—C1—S	124.6 (5)	C12A—C11A—H11B	110.5
C2—C1—S	127.9 (5)	O2—C11A—H11B	110.5
C3—C2—C7	119.2 (6)	H11A—C11A—H11B	108.7
C3—C2—C1	136.8 (6)	O2—C11B—C12B	99 (2)
C7—C2—C1	103.9 (5)	O2—C11B—H11C	111.9
C2—C3—C4	116.7 (6)	C12B—C11B—H11C	111.9
C2—C3—H3	121.7	O2—C11B—H11D	111.9
C4—C3—H3	121.7	C12B—C11B—H11D	111.9
C5—C4—C3	124.1 (6)	H11C—C11B—H11D	109.6
C5—C4—Br	117.4 (5)	C11B—C12B—H12D	109.5
C3—C4—Br	118.4 (5)	C11B—C12B—H12E	109.5
C4—C5—C6	119.5 (6)	H12D—C12B—H12E	109.5
C4—C5—H5	120.2	C11B—C12B—H12F	109.5
C6—C5—H5	120.2	H12D—C12B—H12F	109.5
C7—C6—C5	117.2 (6)	H12E—C12B—H12F	109.5
C7—C6—H6	121.4	C14—C13—S	115.7 (6)
C5—C6—H6	121.4	C14—C13—H13A	108.4
O1—C7—C6	125.8 (6)	S—C13—H13A	108.4
O1—C7—C2	111.0 (5)	C14—C13—H13B	108.4
C6—C7—C2	123.2 (6)	S—C13—H13B	108.4
C1—C8—O1	112.0 (6)	H13A—C13—H13B	107.4
C1—C8—C9	134.0 (6)	C13—C14—H14A	109.5
O1—C8—C9	114.1 (5)	C13—C14—H14B	109.5
C10—C9—C8	112.5 (6)	H14A—C14—H14B	109.5
C10—C9—H9A	109.1	C13—C14—H14C	109.5
C8—C9—H9A	109.1	H14A—C14—H14C	109.5
C10—C9—H9B	109.1	H14B—C14—H14C	109.5
			
O4—S—C1—C8	144.9 (6)	C1—C2—C7—C6	−178.0 (6)
C13—S—C1—C8	−102.2 (6)	C2—C1—C8—O1	0.7 (8)
O4—S—C1—C2	−27.1 (7)	S—C1—C8—O1	−172.7 (4)
C13—S—C1—C2	85.8 (6)	C2—C1—C8—C9	−178.5 (7)
C8—C1—C2—C3	179.7 (7)	S—C1—C8—C9	8.1 (12)
S—C1—C2—C3	−7.2 (12)	C7—O1—C8—C1	−0.6 (7)
C8—C1—C2—C7	−0.6 (7)	C7—O1—C8—C9	178.8 (5)
S—C1—C2—C7	172.5 (5)	C1—C8—C9—C10	76.1 (10)
C7—C2—C3—C4	−0.9 (9)	O1—C8—C9—C10	−103.1 (7)
C1—C2—C3—C4	178.8 (7)	C11B—O2—C10—O3	15.7 (16)
C2—C3—C4—C5	0.5 (10)	C11A—O2—C10—O3	−10.5 (12)
C2—C3—C4—Br	177.8 (5)	C11B—O2—C10—C9	−162.7 (14)
C3—C4—C5—C6	−1.0 (11)	C11A—O2—C10—C9	171.2 (8)
Br—C4—C5—C6	−178.3 (5)	C8—C9—C10—O3	−22.6 (10)
C4—C5—C6—C7	1.7 (10)	C8—C9—C10—O2	155.7 (6)
C8—O1—C7—C6	178.4 (6)	C10—O2—C11A—C12A	179.1 (9)
C8—O1—C7—C2	0.2 (7)	C11B—O2—C11A—C12A	75 (4)
C5—C6—C7—O1	179.9 (6)	C10—O2—C11B—C12B	−126 (2)
C5—C6—C7—C2	−2.1 (10)	C11A—O2—C11B—C12B	−38 (2)
C3—C2—C7—O1	180.0 (5)	O4—S—C13—C14	50.4 (7)
C1—C2—C7—O1	0.2 (7)	C1—S—C13—C14	−61.1 (7)
C3—C2—C7—C6	1.7 (10)		

### Hydrogen-bond geometry (Å, °)

**Table:**

*D*—H···*A*	*D*—H	H···*A*	*D*···*A*	*D*—H···*A*
C11A—H11B···Cg1^i^	0.97	3.06	3.806 (9)	135
C12A—H12B···Cg2^i^	0.96	2.92	3.85 (1)	164
C3—H3···O4^ii^	0.93	2.67	3.556 (8)	160
C5—H5···O3^iii^	0.93	2.67	3.528 (9)	155
C9—H9B···O4^iv^	0.97	2.35	3.277 (9)	161

Symmetry codes: (i) *x*, *y*+1, *z*; (ii) −*x*+1, −*y*, −*z*; (iii) −*x*+1, −*y*, −*z*+1; (iv) −*x*+2, −*y*, −*z*.
